# Light signaling-mediated growth plasticity in Arabidopsis grown under high-temperature conditions

**DOI:** 10.1007/s44154-022-00075-w

**Published:** 2022-12-15

**Authors:** Qi Wang, Ziqiang Zhu

**Affiliations:** College of Life Sciences, Nanjing Normal University, Nanjing, 210023 China

**Keywords:** Phytochrome, PIF4, Thermomorphogenesis, Heat stress, HY5, COP1

## Abstract

Growing concern around global warming has led to an increase in research focused on plant responses to increased temperature. In this review, we highlight recent advances in our understanding of plant adaptation to high ambient temperature and heat stress, emphasizing the roles of plant light signaling in these responses. We summarize how high temperatures regulate plant cotyledon expansion and shoot and root elongation and explain how plants use light signaling to combat severe heat stress. Finally, we discuss several future avenues for this research and identify various unresolved questions within this field.

## Introduction

Most people living in Europe, China, and the US have experienced extreme heat waves over the course of this summer (Witze, 2022). Climate researchers predict that long-lasting heat stress will become increasingly common in the future (Chen et al., 2020) and suggest that even a 1 °C increase in the global average temperature will result in a reduction in global crop yields by 3.1% (in soybean) to 7.4% (in maize) (Zhao et al., 2017). Therefore, in addition to the work being done to control global carbon emissions, we must urgently focus on the production of high-temperature resilient plants to solve the food security issue presented by global warming.

Plants, as sessile organisms, are highly sensitive to increasing temperatures. However, they are adaptive as can be seen when using *Arabidopsis thaliana* as an example. In these plants, increases in temperature from their normal growth (22 °C) to high ambient temperature (28 °C) induce a series of morphological changes (thermomorphogenesis), including hypocotyl elongation, leaf petiole elongation, root elongation, reduced cotyledon expansion, and leaf hyponasty (Casal and Balasubramanian, 2019; Delker et al., 2022; Quint et al., 2016), to reduce temperature stress. Experimental evidence demonstrates that plants use thermomorphogenesis to cool their leaf surface as a means of reducing heat damage (Crawford et al., 2012). However, when temperatures further increase, moving beyond 37 °C, most plants will encounter severe heat stress, which reduces long-term survival because most plants cannot survive long-term exposure to these extreme temperatures (Zhu et al., 2022).

Over the past decade, many well-designed studies have illustrated how plants sense and signal high temperatures to regulate their growth and development. Interestingly, plant photoreceptors and light-signaling components play crucial roles in plant thermomorphogenesis, with several elegant review papers summarizing the interactions between light and temperature (Bian et al., 2022; Ding and Yang, 2022; Jin and Zhu, 2019; Li et al., 2022; Qi et al., 2022). Given this, we have avoided potential content overlap with these reviews by focusing on a discussion of the various interactions between light and temperature and their roles in the regulation of individual plant organ growth. Readers are encouraged to read the other review papers mentioned above for a more nuanced view of the crosstalk between light and high ambient temperature signaling. Please be noted that plants mentioned in this review are *Arabidopsis thaliana* if not specified. The temperature ranges for thermomorphogenesis are 26–30 °C (aka high ambient temperature) and heat stress temperatures are 37–45 °C.

## Light signaling and thermomorphogenesis

Light, the most influential environmental cue in plants, regulates and facilitates photosynthesis and serves as a signal to control plant growth and development at almost all stages of life, ranging from seedling germination to fruit ripening. Plants use various photoreceptors to sense light of various wavelengths, with the best-described systems found in *Arabidopsis thaliana*. UV RESISTANCE LOCUS8 (UVR8) senses short-wavelength UV-B light (280–320 nm) (Podolec et al., 2021), while blue light (390–500 nm) sensing is supported by several different types of blue light photoreceptors. CRYPTOCHROME1 (CRY1) and CRY2 are two of the most important blue light photoreceptors known to control plant hypocotyl elongation, flowering time, and circadian rhythm (Wang and Lin, 2020). From an evolutionary biology perspective, CRYs are the only photoreceptors found in all organisms, ranging from bacteria to humans. Three F-box proteins, ZEITLUPE (ZTL), light/oxygen/voltage (LOV) KELCH PROTEIN2 (LKP2), and FLAVIN-BINDING KELCH REPEAT-BOX1 (FKF1), are also able to directly sense blue light as photoreceptors and regulate floral transition and clock (Ito et al., 2012), while PHOTOTROPIN1 (PHOT1) and PHOT2 blue light photoreceptors, contribute to the phototropic response (Hart and Gardner, 2021). Red and far-red light (600–750 nm) photoreceptors phytochromes are the firstly identified plant photoreceptors. There are five phytochromes in *A. thaliana* (phyA-phyE). Unlike other photoreceptors, phytochromes are found in both active and inactive forms that are reversibly switched under red or far-red light irradiation. In these systems, red light absorption activates the phytochromes (Pfr form) and induces their translocation to the nucleus, where they trigger the red light response. However, far-red light treatment inactivates Pfr, returning them to their inactive form and inhibiting their signaling function (Cheng et al., 2021).

Light signal transduction mechanisms have been largely resolved through extensive genetic screening and protein–protein interaction assays. Interestingly, although individual photoreceptors perceive a specific light spectrum, the molecular basis of their downstream signaling is highly conserved. In general, there are two major paradigms of light signaling. The first model for light signaling relies on protein degradation, with E3 ligase CONSTITUTIVE PHOTOMORPHOGENIC1 (COP1) acting as the central negative regulator of these systems. COP1, initially identified using forward genetic screening in the 1990s (Deng et al., 1991, 1992), directly targets many transcription factors for degradation via the 26S proteasome-mediated protein degradation pathway. Transcription factor ELONGATED HYPOCOTYL5 (HY5) was the first reported COP1 substrate known to directly control the expression of light-responsive genes (Osterlund et al., 2000; Zhang et al., 2011). Light-activated photoreceptors repress COP1 activity to stabilize COP1-targeted transcription factors and elicit a light response. However, the interaction between photoreceptor CRY and COP1 is not light-dependent (Wang et al., 2001; Yang et al., 2000); therefore, there was a large gap in our understanding of this pathway until a series of breakthrough revelations were reported in 2011. COP1 E3 ligase activity is stimulated through interaction with the SUPPRESSOR OF PHYA (SPA) proteins (SPA1-SPA4) (Seo et al., 2003). Chentao Lin’s group and Hong-Quan Yang’s group separately reported that CRYs interact with SPA proteins in a blue light-dependent manner, which further curtails COP1-SPA interactions and reduces COP1 activity (Lian et al., 2011; Liu et al., 2011a; Zuo et al., 2011). Subsequent studies have also demonstrated that phytochromes specifically interact with SPA following red light exposure and function to abrogate COP1-SPA interactions (Lu et al., 2015; Sheerin et al., 2015). These studies clearly illustrate how photoreceptors regulate COP1 activity in a light-dependent manner.

The second paradigm involves interaction between photoreceptors and transcription factors. This mechanism is significant because all photoreceptors, except for the membrane-localized phototropin, translocate or are constitutively located within the nucleus upon light irradiation (Chen et al., 2005; Hiltbrunner et al., 2005; Liu et al., 2022; Qian et al., 2016; Yin et al., 2016; Yu et al., 2007). These receptors then mediate their impact via various PHYTOCHROME INTERACTING FACTORs (PIFs), which were the first reported phytochrome-interacting transcription factors (Ni et al., 1998; 1999). CRY1/CRY2 also interact with PIFs (Ma et al., 2016; Pedmale et al., 2016). In *A. thaliana*, there are seven PIF family members, belonging to the basic helix-loop-helix (bHLH) transcription factor family. These seven PIF proteins play partially redundant roles, although several are also known to perform fairly specific functions under certain conditions (Xu et al., 2015). Most PIF transcription factors are positive regulators of cell elongation that directly bind to auxin biosynthesis-related gene promoters and activate their transcription (Franklin et al., 2011; Sun et al., 2012). Phytochromes interact with PIFs and rapidly trigger PIF protein degradation (Al-Sady et al., 2006; Lorrain et al., 2008; Park et al., 2004; Shen et al., 2008, 2007), while interactions between CRY and PIF result in the inhibition of PIF transcriptional activity (Ma et al., 2016; Pedmale et al., 2016). Photoreceptors are not limited to their interactions with PIF transcription factors but are also known to interact with the majority of transcription factors associated with plant hormone signaling with different regulatory mechanisms. For example, CRY1 and PhyB interact with AUXIN/INDOLE-3-ACETIC ACID-INDUCIBLE (AUX/IAA) proteins (Xu et al., 2018). AUX/IAA proteins are key transcriptional repressors of auxin signaling; these interactions safeguard the stability of AUX/IAA proteins and modulate auxin signaling (Xu et al., 2018). CRY1 and PhyB also interact with AUXIN RESPONSE FACTOR6 (ARF6) and ARF8, regulating their DNA-binding activity and controlling hypocotyl elongation (Mao et al., 2020). UVR8, PhyB, and CRY1 also interact with BRI1-EMS-SUPPRESSOR1 (BES1) to repress hypocotyl elongation via the modulation of brassinosteroid (BR) signaling (Liang et al., 2018; Wang et al., 2018; Wu et al., 2019).

Interestingly, most of these light signaling components also participate in plant responses to high ambient temperature. This is because high ambient temperature stimulates the dark reversion process changing the ratio of active to inactive photoreceptor. PhyB, the first thermosensor identified in *A. thaliana,* is known to rely on changes in the Pr:Pfr ratio to help the plant sense changes in temperature (Jung et al., 2016; Legris et al., 2016). A recent study revealed that phyB undergoes liquid–liquid phase separation (LLPS) under red light and colocalizes with various signaling components, including PIF4, within the phyB condensates. This suggests that the phyB condensates may serve as a hub for signal incorporation. Furthermore, high-temperature treatments reduce phyB LLPS, which is essential for thermomorphogenesis (Chen et al., 2022). High temperatures also modulate the LLPS of EARLY FLOWERING3 (ELF3), a key component in the plant evening complex, which in turn negatively regulates *PIF4* expression and transcriptional activity (Box et al., 2015; Nieto et al., 2015; Nusinow et al., 2011; Raschke et al., 2015). The prion domain (PrD) in ELF3 senses high ambient temperatures and facilitates ELF3 LLPS to inactivate the evening complex (Jung et al., 2020). Phototropins also sense temperature changes, as illustrated in a study of *Marchantia polymorpha*. In this study, the plants were shown to take advantage of temperature-sensitive photoactivated phototropin activity to regulate chloroplast positions for optimized photosynthesis (Fujii et al., 2017).

PIF4 is a central positive regulator of thermomorphogenesis, with plants without PIF4 (*pif4* mutant) known to be largely insensitive to high temperatures at both the phenotype and genome-wide transcriptome levels (Jin et al., 2020; Koini et al., 2009; Kumar et al., 2012). PIF7 interacts with PIF4 and co-regulates gene expression and thermomorphogenesis (Chung et al., 2020; Fiorucci et al., 2020). A recent study argued that PIF7, but not PIF4, is essential for the synergistic effects observed for hypocotyl elongation in response to combining higher ambient temperatures and shade conditions (Burko et al., 2022). Further studies demonstrated that high temperatures enhance *PIF7* mRNA translation because of a change in the conformation of the 5′-untranslated region of *PIF7* mRNA, which increases translation efficiency (Chung et al., 2020). These hairpin structures also exist in many other high-temperature-responsive mRNAs, including *WRKY22* and *HsfA2*, suggesting that this hairpin structure is a conserved thermo-sensing mechanism (Chung et al., 2020).

In addition, high-temperature-triggered hypocotyl elongation is repressed specifically under blue light irradiation. Blue light-activated CRY1 interacts with PIF4 and inhibits its transcriptional activity, which suppresses thermomorphogenesis (Ma et al., 2016). Another recent study reported that CRY2 accumulation is controlled by temperature, and low ambient temperature (16 °C) conditions increase CRY2 protein degradation via its strong interaction with E3 ligase Light-Response Bric-a-Brack/Tramtrack/Broad (LRB) proteins (LRB1/LRB2/LRB3) (Ma et al., 2021). LRBs directly ubiquitinate CRY2 and stimulate protein turnover via the 26S proteasome (Chen et al., 2021; Ma et al., 2021). These results demonstrate that CRYs participate in plant responses to high ambient temperatures.

COP1-SPA1 also positively regulates thermomorphogenesis (Delker et al., 2014; Nieto et al., 2022; Park et al., 2017) because high temperatures stimulate COP1 nuclear localization and facilitate HY5 degradation (Park et al., 2017). HY5 directly represses *PIF4* transcription, and the degradation of HY5 results in the accumulation of *PIF4* mRNA (Delker et al., 2014). HY5 also competes with PIF4 to occupy the PIF4 DNA-binding sites, adding another layer of antagonism to these two transcription factors (Bian et al., 2022; Gangappa and Kumar, 2017). In addition to its interactions with COP1, SPA1 also exerts some Ser/Thr kinase activity (Lee et al., 2020; Paik et al., 2019; Wang et al., 2021a). Thus, SPA1 can directly phosphorylate PIF4, with this activation being essential for PIF4 protein stability. This was confirmed by the fact that the absence of all four SPA proteins (*spaQ* mutants) results in a significant reduction in PIF4 expression. This data also revealed that *spaQ* plants presented with much shorter hypocotyls under high ambient temperature conditions (Lee et al., 2020).

**Fig. 1:**
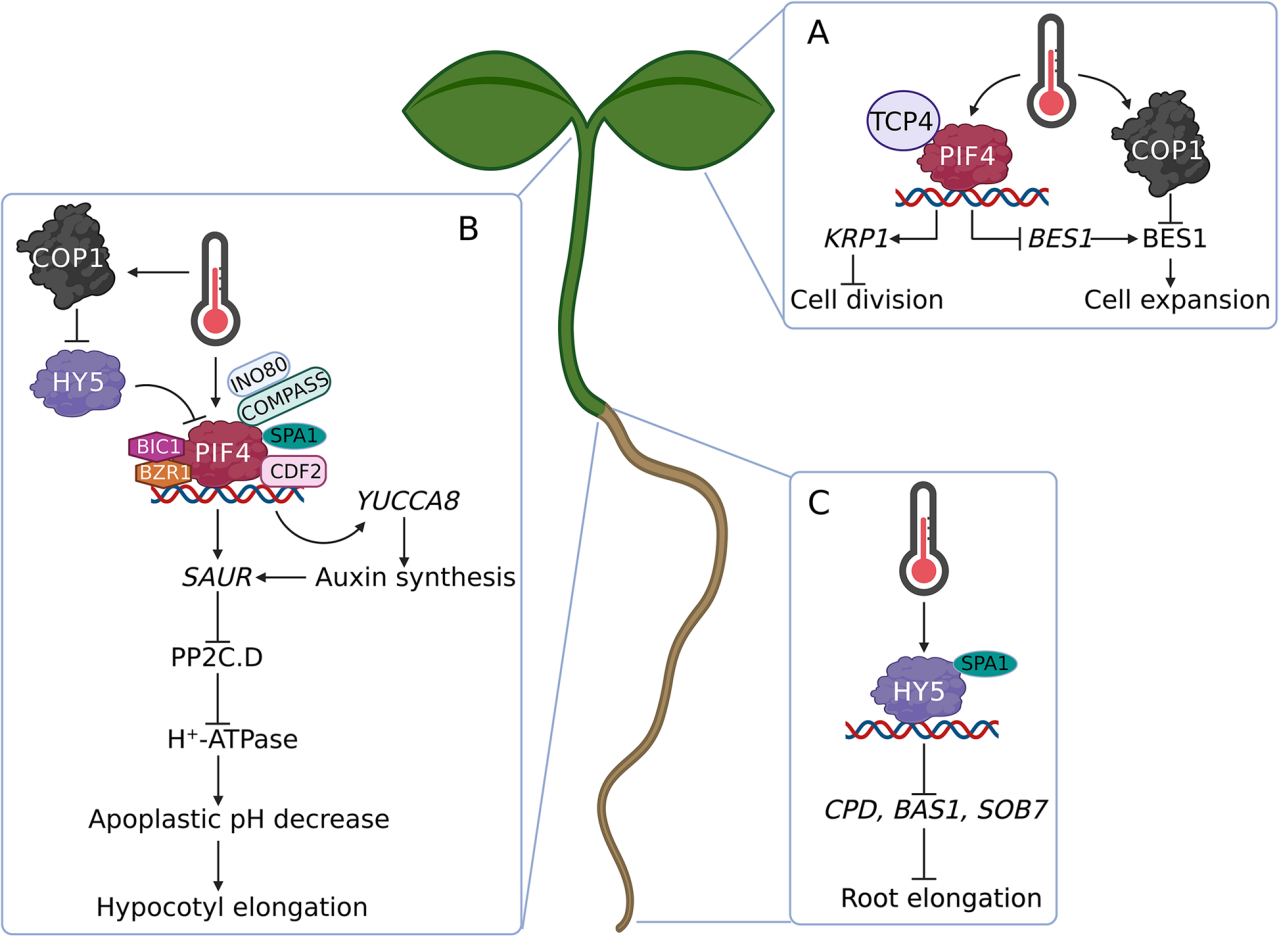
High ambient temperature inhibits plant leaf expansion and promotes cell elongation in both hypocotyl and root. **A** In the leaves, high temperature activated transcription factor PIF4 interacts with TCP4. The TCP4-PIF4 module binds to *KRP1* promoter and induces *KRP1* expression to inhibit cell division. BES1 is a positive regulator for cell expansion. PIF4 directly represses *BES1* transcription. On another side, high temperature increases COP1 nuclear abundances to directly target BES1 protein for degradation. These mechanisms act together to restrict leaf expansion under high ambient temperature. **B** PIF4 plays central roles in the high temperature induced hypocotyl elongation. PIF4 directly binds to the promoter of *YUCCA8* to induce *YUCCA8* expression for boosting auxin biosynthesis. The accumulated auxin further promotes the expression of *SAURs*. PIF4 is also able to induce *SAUR* transcription directly. SAUR proteins interact with PP2C.D enzymes and inhibit their enzymatic activities. The inactivation of PP2C.D further releases their repression on H^+^-ATPase to trigger proton transport into the apoplast. The reduction of apoplastic pH results in cell elongation due to the cell wall loosen and water uptake increase. A lot of co-factors (depicted as INO80-COMPASS, BIC1-BZR1) interact with PIF4 to enhance its transcriptional activities. SPA1 phosphorylates PIF4 to control PIF4 protein stability. The binding of PIF4 with DNA will facilitate other transcription factor (like CDF2) binding to DNA as well. High temperature also stimulates COP1 nucleus localization and enhances HY5 protein turn over. HY5 not only directly represses *PIF4* transcription but also interacts with PIF4 protein to abrogate its transcriptional activity. The removal of HY5 results in the activation of PIF4. **C** High temperature triggers HY5 protein accumulation in roots, which directly controls the expression of brassinosteroid (BR) metabolism genes (*CPD*, *BAS1* and *SOB7*) and root cell elongation. SPA1 also regulates HY5 protein phosphorylation and protein stability

**Fig. 2:**
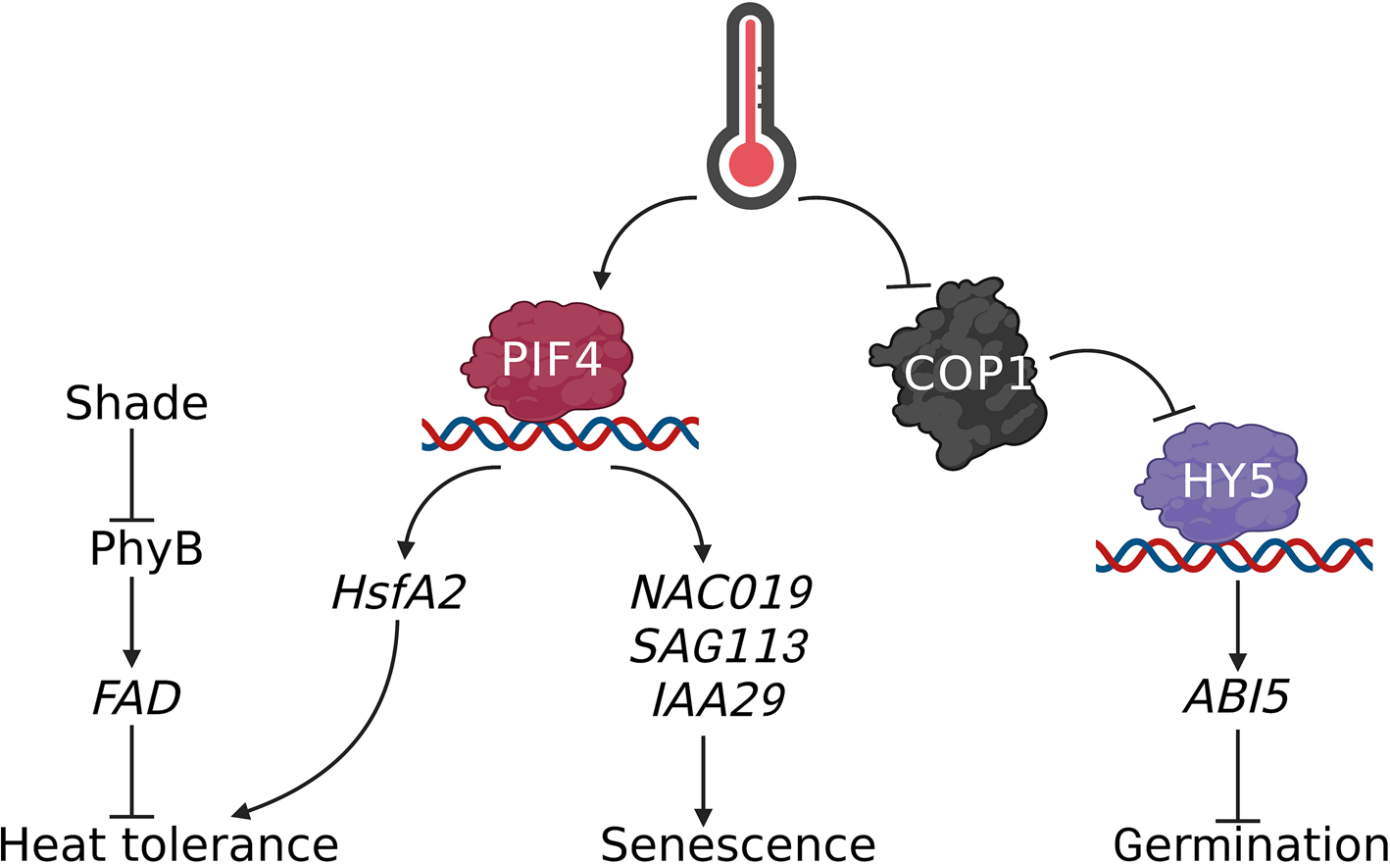
Roles of the major light signaling components in plant heat responses. Light activated transcription factor PIF4, which directly induces *HsfA2* expression to confer seedling heat tolerance. However, PIF4 also triggers senescence associated gene (*NAC019*, *SAG113* and *IAA29*) expressions in adult plants to induce plant senescence. Simulated shade enhances plant heat tolerance through the repression of *FAD* expression to reduce the total unsaturated fatty acids. Heat also inhibits COP1 activity to stabilize HY5, which directly induces *ABI5* expression to repress seed germination

Taken together, these data reveal that light-signaling components are critical players in the high-temperature response for most plants. In the next sections, we describe the roles of various light signaling components in the regulation of plant growth at high ambient temperatures (Fig. 1) and under high-temperature stress (Fig. 2).

## Regulating cotyledon expansion

Cotyledons are specialized organs designed to provide nutrients for seed germination (Chandler, 2008), and both the initiation and outgrowth of these structures are integrated into the embryonic patterning program. In *A. thaliana*, the zygote undergoes cell division to form a radially symmetrical embryo, called a globular embryo, and the cotyledon primordia arise as two distinct bumps from the apex of this structure. These embryos are then converted into one embryo with two planes of bilateral symmetry (Bowman and Floyd, 2008; Chandler et al., 2008; Ito et al., 2011). Previous studies have indicated that the symmetrical positioning of the cotyledon primordia and establishment of cotyledon boundaries correlate with changes in auxin distribution, and treatment with exogenous or polar auxin transport inhibitors results in varying defects across the apical patterning of these embryos, including abnormal positioning and cotyledon fusion (Blilou et al., 2005; Friml et al., 2003; Vieten et al., 2005).

Cotyledon expansion is a major agronomic trait for early seedling vigor (Hahm et al., 2020; Li et al., 2012; Neff and Van Volkenburgh, 1994), known to depend exclusively on cell expansion (Gonzalez et al., 2012; Tsukaya et al., 1994). *A. thaliana* SPATULA (SPT) has been identified as a potent regulator of cotyledon expansion, with *spt* mutants displaying significantly expanded cotyledons with larger pavement cells than the wild-type plants when grown under red-light conditions (Josse et al., 2011; Penfield et al., 2005). Furthermore, cell expansion in plants is often associated with endoreplication, a cell cycle variant during which several rounds of genome duplication occur without subsequent cell division (De Veylder et al., 2011; Marco D'Ario, 2021; Shu et al., 2018). Exogenous nitrate upregulates the CDK-specific inhibitor gene, *LOSS in GIANT CELLS FROM ORGANS* (*LGO*), to increase cotyledon endoreplication and cell size (Moreno et al., 2020). Notably, the bHLH transcription factor CYTOKININ-RESPONSIVE GROWTH REGULATOR (CGK) reportedly promotes cell cycle movement into the S phase, inducing cellular expansion in a ploidy-independent manner in these organs (Park et al., 2021).

High ambient temperatures suppress cotyledon expansion in *A. thaliana* (Delker et al., 2022; Hahm et al., 2020), with increased temperatures activating PIF4 and thus inducing auxin biosynthesis in the cotyledons (Jung et al., 2016; Legris et al., 2016; Procko et al., 2014). Although auxin moves to the hypocotyl to promote cell elongation, excessive accumulation of auxin in the cotyledons inhibits cell expansion (Bellstaedt et al., 2019; Enders et al., 2017). BRI1-EMS-SUPPRESSOR1 (BES1) is a vital transcription factor known to control brassinosteroid-regulated gene expression and promote hypocotyl and cotyledon growth in *A. thaliana* (Nolan et al., 2020). A recent study found that *pif4* mutants presented with elevated *BES1* mRNA levels in cotyledons, indicating that PIF4 likely represses *BES1* expression in cotyledon cells. Further analysis also showed that shade or heat exposure increases PIF4 nuclear localization in cotyledon cells increasing the transcriptional repression of *BES1* and restricting cotyledon expansion (Costigliolo Rojas et al., 2022). In addition, post-transcriptional evaluations reveal that both shade and heat exposure increase COP1 nuclear localization in cotyledon cells, facilitating increased BES1 protein degradation and thereby reducing cotyledon expansion (Costigliolo Rojas et al., 2022). Therefore, the spatial regulation of COP1 and PIF4 nuclear localization in cotyledon cells shapes cotyledon growth plasticity under high ambient temperatures.

As cotyledons and true leaves have similar organ expansion programs and mature morphologies, they can be considered homologous (Chandler, 2008). Thus, it is not surprising that high ambient temperature also reduces the expansion of the true leaf blade (Ibanez et al., 2017; Jin et al., 2011)*.* These observations were supported by a recent report which showed that PIF4/TCP4 suppresses leaf area by inhibiting cell division under high temperatures (Saini et al., 2022). TCP4 is a CINCINNATA (CIN)-like TEOSINTE BRANCHED1/CYCLOIDEA/PCF (TCP) transcription factor, which inhibits cell division by activating cell cycle inhibitor gene *KIP-RELATED PROTEIN1* (*KRP1*) (Schommer et al., 2014). PIF4 physically interacts with TCP4 to promote the expression of *KRP1,* which in turn inhibits cell division, reducing leaf size under high temperatures (Saini et al., 2022).

Taken together, these results reveal that high ambient temperature initially increases auxin biosynthesis in cotyledons by activating PIF4, and excessive residual auxin levels in cotyledons inhibit cell expansion. Second, high ambient temperature downregulates nuclear BES1 levels via PIF4-mediated transcriptional regulation and COP1-mediated post-transcriptional control to suppress cotyledon expansion. Third, high ambient temperatures induce the accumulation of PIF4 in leaf cells to reduce cell division and leaf size in a TCP4-dependent manner (Fig. 1A).

## Regulating hypocotyl elongation

*A. thaliana* hypocotyls elongate in response to increased ambient temperature (Gray et al., 1998), and these elongated hypocotyls are the primary characteristic by which we can recognize thermomorphogenesis. Like in the examples above, PIF4 acts as a central regulator for plant hypocotyl elongation under high ambient temperatures, with these increased temperatures inducing *PIF4* transcription and stabilizing PIF4 protein for its activation (Koini et al., 2009; Qiu, 2020). These activated proteins then directly associate with the *YUCCA8* promoter and induce *YUCCA8* expression (Sun et al., 2012). *YUCCA8,* in turn, encodes a flavin monooxygenase necessary for auxin biosynthesis, which converts indole-3-pyruvic acid (IPA) into indole-3-acetic acid (IAA) (Dai et al., 2013; Mashiguchi et al., 2011; Stepanova et al., 2011; Won et al., 2011). The loss-of-function *yucca8* mutants display short hypocotyls under high ambient temperature, which further suggests that the induction of *YUCCA8* expression is required for thermomorphogenesis (Sun et al., 2012). Interestingly, *YUCCA8* expression is significantly increased in the cotyledons following exposure to high temperatures. Additional experiments demonstrated that hypocotyl elongation is dependent on cotyledon-derived auxins, suggesting that plant cotyledons sense high temperatures and trigger auxin production, which in turn stimulates cell elongation, eventually causing hypocotyl elongation (Bellstaedt et al., 2019). Tissue-specific expression analysis has also shown that epidermal expression of *PIF4* is sufficient to induce *YUCCA8* expression and promote hypocotyl elongation (Kim et al., 2020).

Thus, the PIF4-*YUCCA8* regulatory pathway serves as a hub connecting chromatin remodeling and phytohormone signaling. Recent studies have shown that the INO80 chromatin remodeling complex interacts with PIF4 and mediates H2A.Z eviction, including *YUCCA8,* at PIF4 binding sites (Xue et al., 2021). Interestingly, PIF4 also interacts with BRASSINAZOLE RESISTANT1 (BZR1), a key component in brassinosteroid signaling (Ibanez et al., 2018; Oh et al., 2012). PIF4-BZR1 co-occupies overlapping DNA regions on a genome-wide scale and synergistically regulates hypocotyl elongation (Ibanez et al., 2018; Oh et al., 2012). In addition, another recent study reported that BLUE-LIGHT INHIBITOR OF CRYPTOCHROME1 (BIC1) interacts with BZR1 and PIF4 to co-activate the transcription of their target genes, such as *YUCCA8* (Yang et al., 2021). Interestingly, both BZR1 and BIC1 also positively regulate the expression of *PIF4*, thereby establishing a positive feedback loop to strengthen PIF4 activity (Ibanez et al., 2018; Yang et al., 2021). Further, the PIF4 protein can bind to its promoter to activate *PIF4* transcription, creating a closed positive feedback loop (Zhai et al., 2020). PIF4 binding of the *YUCCA8* promoter also changes the chromatin conformation and enhances the binding of the single zinc-finger transcription factor CYCLING DOF FACTOR2 (CDF2) to the *YUCCA8* promoter (Gao et al., 2022). PIF4 also directly interacts with CDF2, and these two transcription factors cooperate to control hypocotyl elongation (Gao et al., 2022).

In addition, the auxin-mediated acid growth model is widely accepted for explaining cell elongation (Du et al., 2020). Here, accumulated auxin rapidly induces the expression of the SMALL AUXIN UP RNA (SAUR) genes (Stortenbeker and Bemer, 2019); these genes are also regulated via PIF4 interactions with their promotors, which enable increased expression of *SAURs* at high ambient temperatures (Franklin et al., 2011). SAUR19, in turn, interacts with the D-clade type-2C protein phosphatase (PP2C.D) and represses its enzymatic activity (Spartz et al., 2014), reducing plasma membrane H^+^-ATPase activity in these organs (Spartz et al., 2014; Takahashi et al., 2012), which in turn results in increased proton transport into the apoplast. The resulting decrease in apoplast pH loosens the cell wall and increases water uptake and cell elongation (Du et al., 2020). Thus, while the SAUR-PP2C–H^+^-ATPase signaling cascade remains reasonably straightforward, its modulation can facilitate very nuanced changes in its activity, facilitating complex signal crosstalk in response to changes in temperature. For example, ethylene exposure represses thermomorphogenesis through its key transcription factor, ETHYLENE INSENSITIVE3 (EIN3). Although EIN3 does not directly interact with PIF4, EIN3 does activate the expression of PP2C.D (*ARABIDOPSIS PP2C CLADE D7*, *APD7*), repressing H^+^-ATPase activity (Kim et al., 2021) and facilitating much more complex regulation of these response systems.

Thus taken together, we can conclude that high ambient temperatures initially activate PIF4 within the epidermis, inducing auxin biosynthesis. Auxin is then moved to the hypocotyl, where it acidifies the cell walls, inducing cellular elongation (Fig. 1B).

## Regulating root elongation

High ambient temperature not only affects the growth and development of the aerial portions of plants but also their roots (Martins et al., 2017). However, unlike the extensive studies described for hypocotyl elongation, the root response to high ambient temperatures is much less well-defined (CFF et al., 2021). Similar to the promotion of hypocotyl elongation, high ambient temperatures stimulate root elongation under continuous light or long-day conditions (root thermomorphogenesis) (Borniego et al., 2022; Gaillochet et al., 2020; Lee et al., 2021; Martins et al., 2017). A detailed analysis has also demonstrated that high temperatures trigger cell elongation, but not division, to promote root elongation (Martins et al., 2017), and there are several interesting differences between hypocotyl and root thermomorphogenesis.

First, in contrast to the critical role of YUCCA8-dependent auxin biosynthesis in the regulation of hypocotyl elongation, high-temperature treatment does not significantly alter *YUCCA8* expression, auxin marker gene expression, or auxin accumulation (Martins et al., 2017) in the roots. Auxin receptor mutants exhibit identical root elongation phenotypes to wild-type plants, suggesting that auxin biosynthesis is not involved in root thermomorphogenesis (Martins et al., 2017). Second, the thermosensors (phyB, ELF3, and PIF7) identified in hypocotyl thermomorphogenesis are not required for root thermomorphogenesis. This finding was exemplified by several studies that showed that even in *phyB* and *elf3* mutants, the plants still produced normal-length roots (Borniego et al., 2022; Gaillochet et al., 2020). High-temperature treatment also did not reduce phyB nuclear body size, further indicating that phyB is not a thermosensor in root thermomorphogenesis. In addition, while high temperatures enhance *PIF7* translation during hypocotyl thermomorphogenesis, there is no increase in its accumulation in the roots (Borniego et al., 2022). Therefore, we can assume that none of these three major thermosensors fulfill these roles in the roots. Third, PIF4 and its homologs are dispensable for root thermomorphogenesis, with the root elongation phenotypes in *PIF4* loss-of-function mutants (*pif4*) and *pifQ* (*pif1 pif3 pif4 pif5*) quadruple mutants shown to be similar to those of wild-type plants (Gaillochet et al., 2020; Lee et al., 2021). In fact, *PIF4* is primarily expressed in the aerial parts of the plant but not in the roots (Lee et al., 2021). Taken together, these results suggest that plant root responses to high ambient temperatures are distinct from that of their hypocotyls.

This was further supported by two independent studies, which reveal that HY5 is necessary for plant root thermomorphogenesis. They showed that *hy5* mutants display short root phenotypes under high ambient temperatures (Gaillochet et al., 2020; Lee et al., 2021), and *HY5* is predominantly expressed in the roots. These experiments also show that high temperatures induce *HY5* transcription in the roots (Lee et al., 2021). In addition, subsequent experiments using cotyledon-specific promoter *CAB3* to express the HY5 protein tagged with HA-YFP-HA (DOF-HY5) to restrict HY5 protein movement from shoot to root, results in the *hy5* mutant background (*pCAB3*:*DOF-HY5*/*hy5*) not being able to rescue the *hy5* short root phenotypes under high ambient temperature (Gaillochet et al., 2020). Transcriptomic analysis revealed that high temperatures elicit distinct differentially expressed gene profiles, and unlike the regulation of growth-related genes in hypocotyls, high-temperature exposure preferentially modulates metabolism-related genes in root samples (Gaillochet et al., 2020; Lee et al., 2021). For example, HY5 directly controls the expression of several genes (*CPD*, *BAS1*, and *SOB7*) involved in brassinosteroid metabolism, allowing this factor to modulate brassinosteroid signaling (Lee et al., 2021) involved in root thermomorphogenesis (Martins et al., 2017) (Fig. 1C).

Furthermore, SPA proteins phosphorylate PIF4, and this phosphorylation is required for PIF4 stability and hypocotyl thermomorphogenesis (Lee et al., 2020). Interestingly, this scenario recapitulates during root thermomorphogenesis, where SPA1 phosphorylates HY5 at serine 36 (S36) (Wang et al., 2021a). Overexpression of phosphor-null HY5 (S36A) in the *hy5* mutants resulted in an inability to rescue the *hy5* mutant root phenotypes under high ambient temperature, while overexpression of phospho-mimic HY5 (S36D) in the *hy5* background mediated clear root elongation (Lee et al., 2021). Further immunoblot studies showed that HY5 phosphorylation was positively correlated with its protein stability in the roots. Thus, it was not surprising that *spaQ* mutants retain their short root phenotype under high ambient temperatures (Lee et al., 2021). Thus, we can conclude that SPA proteins are necessary for both hypocotyl and root thermomorphogenesis.

## Roles in heat stress responses

The previous sections focused on plant responses to high ambient temperatures, such as temperatures that trigger thermomorphogenesis in *A. thaliana* (26–30 °C). However, when plants grow in temperatures above this limit, they no longer experience thermomorphogenesis but rather enter heat stress. Heat stress has many adverse effects on plant growth and development, including inhibition of germination, growth, reduced fertility, and cell death (heat stress responses) (Ohama et al., 2017). Heat shock transcription factors (Hsf) are rapidly induced in response to heat treatment and are known to function as master regulators of various *HEAT SHOCK PROTEINs* (*HSPs*) (Charng et al., 2007; Liu et al., 2011b; Ohama et al., 2017). HSPs are well-known molecular chaperones that facilitate heat-induced refolding of misfolded proteins (Jacob et al., 2017). A recent study revealed a mobile signal underlying the plant heat response, with heat exposure initially triggering nitric oxygen (NO) production at the apex of the inflorescence. This accumulated NO then interacts with glutathione to form S-nitrosoglutathione (GSNO), which then rapidly moves from the shoot to the root and promotes the production of transcription factor GT-1 S-nitrosylation. S-nitrosylated GT-1 then binds to additional NO-responsive elements in the *HsfA2* promoter and induces *HsfA2* expression, enhancing plant heat tolerance (He et al., 2022). This elegant study not only described the potential heat-sensing organs in plants but also reported a mobile signal for transmitting heat signals from the shoot to the root.

In addition, since PIF4 is a central regulator of thermomorphogenesis, many studies have evaluated its role in the heat stress response. These investigations revealed that similar to high ambient temperatures, heat treatment induces *PIF4* transcription and stabilizes the PIF4 protein (Yang et al., 2022). Phenotypic observations (4-day-old etiolated seedlings grown at 22 °C and then placed at 45 °C for 1 h before being recovered at 22 °C for 3 days) illustrate that *pif4* mutants are less tolerant of heat treatment, while plants overexpressing *PIF4* show enhanced heat tolerance. Further studies have found that PIF4 directly binds to the *HsfA2* promoter and induces *HsfA2* expression, increasing heat tolerance (Yang et al., 2022). However, another study in adult plants demonstrated that PIF4 acts as a positive regulator of plant senescence at high temperatures (Li et al., 2021). One such study used 3-week-old plants treated at 42 °C for 5 h and then recovered for 3 days for observation and revealed that all of these plants started to produce obvious senescent phenotypes (Li et al., 2021). Additional evaluations revealed clear reductions in chlorophyll content in the wild-type plants following heat stress but significant increases in chlorophyll in *pif4* mutants (Li et al., 2021). Consistent with the mutant phenotypes, chlorophyll content was greatly reduced in *PIF4* overexpressing lines, and other data revealed that PIF4 directly binds to the promoter of several senescence-related genes (*NAC019*, *SAG113*, and *IAA29*), inducing their transcription and supporting the transition to senescence in response to heat treatment (Li et al., 2021). Although these studies suggest different roles for PIF4, both sets of results further confirm that PIF4-mediated transcriptional regulation is a critical factor in plant heat responses.

Light conditions also affect plant heat-stress responses. For example, comparisons of white light and shade (low-red/far-red ratios) growth conditions reveal a clear increase in heat shock (45 °C for 45 min) tolerance in the latter group (Arico et al., 2019). PhyB mutants consistently demonstrate enhanced heat tolerance, even under white light conditions (Arico et al., 2019; Song et al., 2017), and the expression levels of fatty acid desaturase (FAD) genes decreased in the shade, suggesting that this may contribute to the reduction in total unsaturated fatty acids and increased membrane stability in these plants (Arico et al., 2019).

In addition, the COP1-HY5 regulatory module also participates in the regulation of seed germination under heat stress, where ABA-INSENSITIVE5 (ABI5) acts as the key transcription factor in the abscisic acid (ABA) signaling pathway, which, in turn, regulates seed germination. Heat promotes COP1 movement from the nucleus to the cytoplasm, increasing HY5 stability and promoting its interactions with the *ABI5* promoter, inducing its expression and increasing the inhibition of seed germination (Chen et al., 2019).

Taken together, these results show that light signaling components are also critical to the heat stress response in plants (Fig. 2).

## Perspectives

Although we have achieved many milestones in understanding the high-temperature signaling response in plants, several unexplored phenomena need to be studied. These include:

1. Evaluation of the precise temperature sensing mechanisms. This is because plants tend to reduce growth in response to lower temperatures following chilling or freezing stress, albeit to different degrees, but increase their growth in response to higher ambient temperatures (26–30 °C; Phase 1), eventually entering restricted growth when temperatures reach the high-temperature stress threshold (Phase 2). We do not know how a plant measures these temperature changes or makes contrasting decisions to enter either of these two phases. Previous studies have focused only on phase 1 or phase 2, and further studies need to focus on their cross-over and simultaneous induction, with initial investigations focusing on known photoreceptors, including PhyB.
2. The roles of PIF4 in different developmental stage and temperature range are deserved to be further investigated. As we described, PIF4 positively regulates cell elongation in hypocotyl cells but negatively controls cell expansion in cotyledon cells under high ambient temperatures. Moreover, PIF4 enhances plant heat stress tolerance in seedlings but triggers senescence in adult plants. Therefore, we assume that the function of PIF4 varies according to distinct stages. The recently popular single cell analysis may contribute to a detailed understanding of PIF4 in a more spatial and temporal way.
3. The mechanisms underlying plant root thermomorphogenesis are not well understood. Although HY5-mediated signaling is required for root elongation at high ambient temperatures, other mechanisms are worthy of further study. This is important because we know that detached roots can elongate under high ambient temperatures, even in the absence of cotyledons and hypocotyls (Bellstaedt et al., 2019), indicating that plant roots can sense temperature without the need for various aerial components of these organisms. In fact, currently reported thermosensors (phyB, ELF3, and PIF7) do not participate in root thermomorphogenesis (Borniego et al., 2022), suggesting that the identification of root thermosensors may be a particularly fruitful avenue of investigation.
4. The LLPS of thermosensors, phyB and ELF3, suggests that high temperature-induced condensates are a common regulatory mechanism in plants, facilitating their nuanced response to changes in temperature. Since CRY2 also undergoes LLPS in response to blue light and co-condense with m^6^A writer proteins to regulate N^6^-methyladenosine modification and circadian rhythm (Wang et al., 2021b), the LLPS status of blue-light-activated CRY2 under high ambient temperatures deserves further exploration.
5. Finally, although we clearly understand these processes in Arabidopsis, there is an urgent need to evaluate its translational value in other plants to transfer this knowledge to the cultivation of food and fruit crops and increase their high-temperature tolerance.

## Data Availability

Not applicable.

## Abbreviations

UVR8: UV RESISTANCE LOCUS8
CRY1: CRYPTOCHROME1
ZTL: ZEITLUPE
LKP2: Light/oxygen/voltage (LOV) KELCH PROTEIN2
FKF1: FLAVIN-BINDING KELCH REPEAT-BOX1
PHOT1: PHOTOTROPIN1
COP1: CONSTITUTIVE PHOTOMORPHOGENIC1
HY5: ELONGATED HYPOCOTYL5
SPA: SUPPRESSOR OF PHYA
PIF4: PHYTOCHROME INTERACTING FACTOR4
AUX/IAA proteins: AUXIN/INDOLE-3-ACETIC ACID-INDUCIBLE proteins
ARF6: AUXIN RESPONSE FACTOR6
ELF3: EARLY FLOWERING3
LRB1: Light-Response Bric-a-Brack/Tramtrack/Broad protein1
BES1: BRI1-EMS-SUPPRESSOR1
BZR1: BRASSINAZOLE RESISTANT1
TCP4: CINCINNATA (CIN)-like TEOSINTE BRANCHED1/CYCLOIDEA/PCF (TCP) transcription factor4
KRP1: KIP-RELATED PROTEIN1
BIC1: BLUE-LIGHT INHIBITOR OF CRYPTOCHROME1
SAUR: SMALL AUXIN UP RNA
EIN3: ETHYLENE INSENSITIVE3
APD7: ARABIDOPSIS PP2C CLADE D7
HSP: HEAT SHOCK PROTEIN
